# Smartphone-Based Bioelectrical Impedance Analysis Devices for Daily Obesity Management

**DOI:** 10.3390/s150922151

**Published:** 2015-09-02

**Authors:** Ahyoung Choi, Justin Younghyun Kim, Seongwook Jo, Jae Hwan Jee, Steven B. Heymsfield, Yusuf A. Bhagat, Insoo Kim, Jaegeol Cho

**Affiliations:** 1 DMC R&D Center, Samsung Electronics, Suwon 16677, Gyeonggi, Korea; E-Mails: ay12.choi@samsung.com (A.C.); vine.kim@samsung.com (J.Y.K.); sw0326.jo@samsung.com (S.J.); 2 Center for Health Promotion, Samsung Medical Center, Seoul 06351, Korea; E-Mail: jaehwan.jee@samsung.com; 3 Pennington Biomedical Research Center, Baton Rouge, LA 70808, USA; E-Mail: steven.heymsfield@pbrc.edu; 4 Samsung Research America, Richardson, TX 75082, USA; E-Mails: y.bhagat@samsung.com (Y.A.B.); insoo3.kim@samsung.com (I.K.)

**Keywords:** body composition analysis, mobile health, obesity management

## Abstract

Current bioelectric impedance analysis (BIA) systems are often large, cumbersome devices which require strict electrode placement on the user, thus inhibiting mobile capabilities. In this work, we developed a handheld BIA device that measures impedance from multiple frequencies (5 kHz~200 kHz) with four contact electrodes and evaluated the BIA device against standard body composition analysis systems: a dual-energy X-ray absorptiometry (DXA) system (GE Lunar Prodigy, GE Healthcare, Buckinghamshire, UK) and a whole-body BIA system (InBody S10, InBody, Co. Ltd, Seoul, Korea). In the study, 568 healthy participants, varying widely in body mass index, age, and gender, were recruited at two research centers: the Samsung Medical Center (SMC) in South Korea and the Pennington Biomedical Research Center (PBRC) in the United States. From the measured impedance data, we analyzed individual body fat and skeletal muscle mass by applying linear regression analysis against target reference data. Results indicated strong correlations of impedance measurements between the prototype pathways and corresponding InBody S10 electrical pathways (R = 0.93, *p* < 0.0001). Additionally, body fat estimates from DXA did not yield significant differences (*p* > 0.728 (paired *t*-test), DXA mean body fat 29.45 ± 10.77 kg, estimated body fat 29.52 ± 12.53 kg). Thus, this portable BIA system shows a promising ability to estimate an individual’s body composition that is comparable to large stationary BIA systems.

## 1. Introduction

Consumer interest towards personalized health, including fitness and weight management, is increasing. With respect to weight control, body composition data such as fat, lean soft tissue (LST), total body water, and skeletal muscle mass bear significant importance in understanding total body energy balance by quantifying the amount of fat to be decreased and muscle to be increased. Typically body composition is measured on the basis of computerized axial tomography scanning (CT scan), magnetic resonance imaging (MRI), dual-energy X-ray absorptiometry (DXA), caliper testing, or body impedance analysis (BIA) devices. The majority of these systems are intended for medical use owing to limitations such as exposure to Gamma radiation (x-rays) and the overall size of the modality, except in the cases of BIA and caliper measurements. Calipers are convenient, but typical skin-fold testing with calipers requires trained experts. Typical stationary eight-electrode BIA systems require strict electrode placement on the user, thus inhibiting mobility. Recently, some manufacturers have released scale-type and portable-type body fat measurement devices, but the size of such devices remains large relative to the level of portability needed, which could be obtained from smaller form factor mobile devices (e.g., Omron’s portable body fat measurement device (HBF-306): 22.86 cm × 5.08 cm × 15.24 cm) [1,2,3]. Further, current scale and portable-type devices also have limitations when measuring whole body impedance because of weak electrical pathway configurations or their inability to measure impedance at multiple frequencies. To guarantee repeatability of impedance measurement and reliability of body fat estimation in different situations, impedance measurements at multiple frequencies were proposed [4]. 

Our purpose was to develop miniature BIA systems that include small, portable, and wireless contact electrodes incorporated into smartphones, wristbands, and wearable electronics. The sensing devices were each equipped with four electrodes: one pair of electrodes to deliver current and another pair to that act as voltage sources. The sizes and locations of the electrodes were optimized to be independent of individual physical conditions such as skin hydration status, contact resistance, and physical force. Such factors have previously been shown to result in the aforementioned discrepancies in BIA studies. Each system measures impedance at multiple frequencies for improved accuracy: 20 kHz, 50 kHz, and 200 kHz. The devices provide body composition assessments such as body fat and skeletal muscle mass by linear regression. We evaluated three prototype pathways in a large healthy population against a commercial adhesive-electrode BIA system and a conventional body composition device such as DXA. 

Body fat estimation was performed by applying regression analysis as previously reported [5,6,7,8,9,10,11,12,13,14] on the basis of measurements by stationary BIA systems. Typically, body fat was estimated from variables such as body weight and estimated Fat Free Mass (FFM). FFM was estimated by multiple linear regression methods using the target FFM from DXA as a criterion standard. The skeletal muscle mass was calculated by linear regression with the target soft lean mass from DXA. Based on previous reports, a myriad of different BIA equations were derived by empirical regression models computed from impedance and the users’ personal profile information including age, gender, and height from large population samples [6]. However, such BIA equations are limited in that they can only be effectively applied to whole-body and stationary BIA systems. Thus in this work, we developed a BIA algorithm to estimate whole body composition by measuring hand-to-hand (or halfway hand-to-leg) impedance with small and portable BIA systems. We collected data from various population samples by accounting for age, gender, race and BMI to develop a reliable BIA algorithm which can be applied to the general population regardless of varying demographics or physical characteristics. Our hypotheses were: (1) that impedance values derived from the prototype devices would be strongly correlated to those measured from the corresponding electrical pathways, as well as the whole body-electrical circuit measured using a clinical grade BIA system and (2) that the impedance index (height^2^/impedance) from the proposed devices and LST as measured by DXA would be strongly correlated. We further aimed to use linear regression modeling to compute reliable body fat and skeletal muscle mass estimates comparable with DXA body composition results. We introduce our prototypes and experimental results as follows: System configuration of proposed prototypes, experimental protocol and subject statistics are indicated in Section 2. Experimental results are described in Section 3. Section 4 concludes this manuscript by presenting main findings and future works. 

## 2. Experimental Section

### 2.1. System Configuration

**Figure 1:**
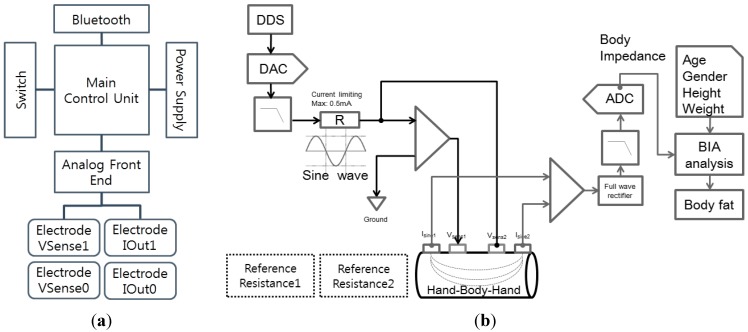
Hardware block diagram of (**a**) overall system and (**b**) the analog front end.

The overall system consists of a smartphone cover-type sensing interface to monitor impedance and a mobile application inside the smartphone to process and display the result. The sensing interface includes sensing electrodes for data acquisition, an analog front end and main control unit for processing data, Bluetooth for transmitting data, and a power supply as shown in Figure 1a. 

The main control unit controls the analog-front-end, a Bluetooth module, the power, and the switch. The analog-front-end is connected to four electrodes: two current-sourcing electrodes for current generation at multiple frequencies and two voltage-sensing electrodes for impedance measurement as shown in Figure 1b. The current source delivers a 500 µA sinusoidal current to the human body at multiple frequencies between 5 kHz and 200 kHz. The voltage electrodes sense voltage levels between two distinct positions and this voltage information is acquired from the ADC module. Acquired voltage values are converted to impedance values by checking the reference resistor impedance. We measured voltage values from reference resistances of 300 Ohms and 1000 Ohms during the initialization step. Body impedance data are estimated by a relative comparison of reference resistance voltages. 

**Figure 2:**
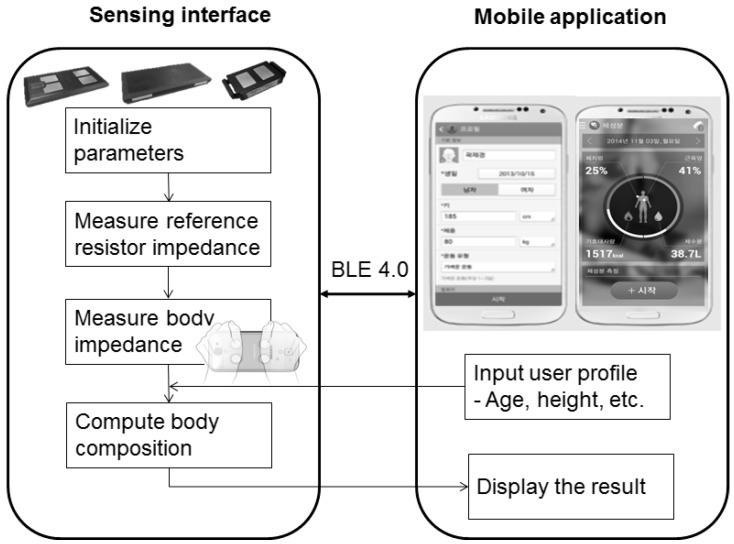
Overall process flow.

Impedance data are transferred to a smartphone for analysis along with user profile information such as age, height, weight, gender, and race. Based on the user profile, the system computes and displays body fat and skeletal muscle mass as shown in Figure 2. Body composition equations are derived from multiple linear regression, which fits to the target value after feature selection. 

### 2.2. Experimental Protocol

The study in the United States was approved by the PBRC Institutional Review Board (IRB) (#2014-005-PBRC SAMSUNG) and was registered in ClinicalTrials.gov (NCT02115425). The study in Korea was approved by the SMC IRB (SMC 2014-03-0630993) and was approved by Ministry of Food and Drug Safety for clinical test (A30320.02). This study protocol was approximately 90 min long. Upon arrival, a researcher explained the study purpose and protocol. Participants completed a consent form if they agreed to participate in the study. They then submitted a preliminary survey which included general user information such as age, gender, race, and medical history, exercise habits, and current medication. Trained experts then performed a physical examination to measure height, weight, wrist, and hip circumference. Vital signs were also measured, consisting of blood pressure, heart rate, skin temperature, and pregnancy tests for female subjects. Extreme outlying values from these parameters were considered to be exclusion criteria for the study. After screening, subjects were transferred to the DXA scanner where they laid down in a supine position for about 10 min. Following completion of the DXA measurements, measurements with the prototype BIA devices were made. The BIA devices measure upper or half-body impedance at each of the three frequencies for about 6 s while subjects maintain arm position at 90° with respect to the torso. Thereafter, impedance and body composition data were measured using other commercial BIA devices, Inbody S10 (InBody Co. Ltd., South Korea) and HBF-214/HBF-370 (Omron, Japan), while subjects continued to maintain a recumbent position on the scanner platform. In each test, 3 measurements were made and the results were averaged for further analysis. 

**Figure 3:**
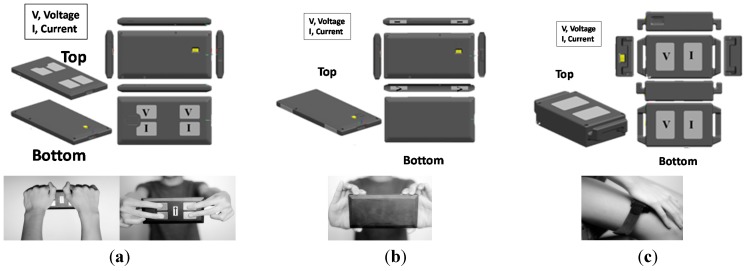
Three pathways of BIA devices (**a**) Palm-Palm (PP) type; (**b**) Finger-Finger (FF) type; and (**c**) Palm-Knee (PK) type

For this study, three different types of wireless BIA devices were developed to address various anatomical pathways: palm-to-palm (PP), finger-to-finger (FF), and palm-to-knee (PK) as shown in Figure 3. In the PP device, the four electrodes (each 20 × 30 mm^2^) were housed on the top surface of the cover. This device ideally measures impedance along the PP pathway in a standing posture. We evaluated the PP device with two different positions: holding the device using one’s palms, PP-P1 (Figure 3a, left), and holding the device using one’s fingers, PP-P2 (Figure 3a, right). For the FF device (Figure 3b), the four electrodes (each 12.5 × 6 mm^2^) are positioned on each lateral edge, permitting four finger-contact points. The PK device (Figure 3c) with four electrodes (each 20 × 30 mm^2^) was designed to measure segmental BIA. It delivers current from the left (or right) hand to the left (or right) knee. PP and FF devices were designed to measure impedance of the upper body, while the PK device measures impedance of the half-body (left arm to leg, or right arm to leg). To determine the optimal electrode surface area, we empirically tested the size of electrodes that showed minimal effect on impedance when pressure and contact was maintained. 

The InBody S10 device measures impedance, resistance, and reactance at six frequencies (1, 5, 50, 250, 500, and 1000 KHz). An 8-point adhesive electrode design is used to evaluate five body segments (right arm, left arm, trunk, right leg, and left leg) in lying, standing with arms at 0°, and standing with arms at 90° positions. Since the PP prototype measures the “arm-arm” pathway, we used the corresponding IB right-to-left-arm pathway for comparative purposes. Similarly, as the PK prototype measures the left or right “palm-knee” pathway, this permitted a comparative analysis with the corresponding segmental BIA pathway from InBody S10. For conventional measurements, we used a GE Lunar Prodigy DXA system with enCORE 2008 software (GE Healthcare, UK). Calibrations were completed daily according to manufacturer specifications. The system software provided estimates of three components: fat, LST, and bone mineral content (BMC), for the whole body, head, arms, trunk, and legs. The coefficient of variation for repeated measurements on this system was between 0.26%–0.29%.

### 2.3. Subject Statistics and Analysis Method

We recruited 568 voluntary participants over two months at two research centers; 256 participants were recruited at SMC, and 312 participants were recruited at PBRC. Participants’ statistics and demographics are shown in Table 1 and Table 2, respectively. Subjects were of Asian, Caucasian, and African American descent. Participants were healthy volunteers over 18 years old, with the mean age being 42.8 ± 17.0. They were stratified across equally distributed age and BMI corresponding to underweight (<18.5 kg/m^2^ for all races), normal (18.5–24.9 kg/m^2^ for Caucasians (C) and African Americans (AA), 18.5–22.9 kg/m^2^ for Asians (A)), overweight (25.0–29.9 kg/m^2^ for C and AA, 23.0–25.0 kg/m^2^ for A) and obese (>30 kg/m^2^ for C and AA, >25.0 kg/m^2^ for A). We further defined subgroups including three age groups (19~39 year-old, 40~59 year-old and 60~79 year-old), two gender groups (male and female), and three BMI groups (underweight and normal, overweight and obese). We attempted to collect similar data across each subgroup. Some data were excluded during screening in cases of early termination due to subject discomfort and withdrawal of consent (6.2%). Subjects who were pregnant, presented with electrical implants such as cardiac pacemakers, or were taking medications for over a one-week period due to an underlying chronic or acute disease were excluded from the study. Body builders were also excluded on the basis that their advanced musculature is not representative of the average muscle composition pertaining to the general public [15,16]. The factors that influenced the 6.2% withdrawal rate in this study were subject discomfort in the course of measurements, dry hands resulting in poor electrode contact in some subjects, and unknown pregnancies in some female subjects which led to their withdrawal from testing. Two hours before participating in the study, subjects were restricted from drinking liquids, eating food, taking hot baths, and engaging in heavy exercise.

**Table 1:** Subject statistics.

Total N		Asian	Caucasian	African American	Total
		258	232	78	568
Basic statistics	Age(year)	47.7 ± 15.1	39.6 ± 17.1	41.4 ± 14.3	42.8 ± 17
	Height(cm)	164.5 ± 8.8	168.3 ± 8.5	165.4 ± 8.1	166.8 ± 8.9
	Weight(kg)	64.7 ± 11.0	73.3 ± 17.3	83.3 ± 18.8	71.3 ± 16.5
	Wrist circumstance(cm)	83.0 ± 8.6	91.2 ± 15.5	97.9 ± 16.8	89.5 ± 43.8
	Hip circumstance(cm)	96.5 ± 6.1	99.7 ± 13.5	108.7 ± 13.7	99.1 ± 11.2

All values are Mean ± SD.

**Table 2:** Subject numbers of subgroup.

		Asian		Caucasian		African American		Total
		Male	Female	Male	Female	Male	Female	
Age group	19~39	46	43	65	77	10	25	266
		(8%)	(8%)	(11%)	(14%)	(2%)	(4%)	(47%)
	40~59	42	42	17	40	7	19	167
		(7%)	(7%)	(3%)	(7%)	(1%)	(3%)	(29%)
	60~79	42	43	10	23	6	11	135
		(7%)	(8%)	(2%)	(4%)	(1%)	(2%)	(24%)
	Total	130	128	92	140	23	55	568
		(23%)	(23%)	(16%)	(25%)	(4%)	(10%)	(100%)
BMI group	Normal/	43	63	36	87	6	12	247
	Underweight	(8%)	(11%)	(6%)	(15%)	(1%)	(2%)	(43%)
	Overweight	39	36	32	28	8	13	156
		(7%)	(6%)	(6%)	(5%)	(1%)	(2%)	(27%)
	Obesity	48	27	24	25	9	30	163
		(8%)	(5%)	(4%)	(4%)	(2%)	(5%)	(29%)
	Total	130	128	92	140	23	55	568
		(23%)	(23%)	(16%)	(25%)	(4%)	(10%)	(100%)

All percentage values in parenthesis are rounded to the first decimal place.

Our analysis included data collection, outlier removal, input variable selection and linear regression. Outliers greater than 2 standard deviations (±2 z) compared to mean values were removed which resulted in two outliers from the SMC data set, and twenty two outliers from the PBRC data set. In this study, distribution of the impedance data is slightly skewed (0.266) and it is closer to lognormal distribution. Based on previous reports, we used ±2 z stringent outlier cutoff point to obtain the zero skewness data set [17]. We then selected features including age, gender, height, weight, race, BMI, exercise level [18], and impedance index by examining various combinations of features. These combinations of input variables showed varying performance levels by applying step-wise regression forward selection with alpha set at 0.5 (Table 3). A four-input variable condition with weight, sex, impedance index and height yielded a regression coefficient of 91.1%, whereas a six-input variable case with weight, sex, impedance index, height, age and race, yielded an R^2^ of 91.6% (Table 3). The target data comprising LST and FFM were derived from DXA body composition results. Hence, we concluded that four input variables were sufficient to estimate body fat and skeletal muscle mass, which were applied to obtain the skeletal muscle and FFM equations for this study. The body fat equation is derived from the difference between body weight and the estimated fat-free mass (FFM) defined as mass comprising muscle, water and bone; the FFM is estimated by linear regression with target FFM data from DXA. Skeletal muscle mass is also computed by the linear regression model using the target soft lean mass data from DXA. Multiple frequency impedance values are used to improve measurement accuracy. The impedance data were ignored or updated if there were different patterns of impedance distribution obtained through the multiple frequencies. 

**Table 3:** Feature combination.

Case **	1	2	3	4	5	6
S	7.65	4.16	3.43	3.17	3.12	3.06
R^2^	55.45	86.86	91.07	92.37	92.66	92.91
Mallows Cp	2830.4	456.7	140.5	44.1	24.3	7.0
PRESS	31869.8	9440.11	7455.04	6318.98	6137.65	5957.88
R^2^(pred)	55.1	86.7	89.5	91.1	91.35	91.61

S: Square root of MSE (Mean Square Error), R^2^: Regression coefficient, Mallows Cp: Assessment of fit of a regression model by error sum of squares, PRESS: Predicted residual sum of squares; ** Each case number indicates; 1: Weight, 2: Sex, 3: Impedance Index, 4: Height, 5: Age and 6: Race.

## 3. Results and Discussion

**Figure 4:**
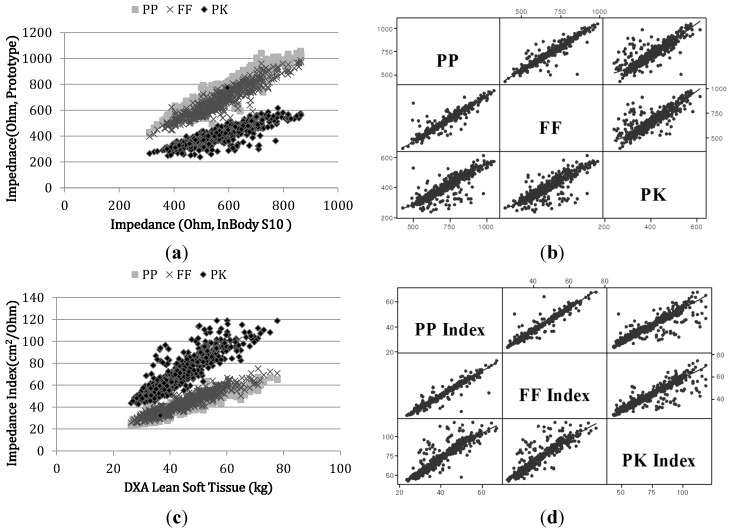
Scatterplots of impedance measured by reference devices vs. prototype devices. (**a**) Impedance scatter plot of the three pathways from each prototype device and the corresponding InBody pathways; (**b**) matrix plots of impedance between each pair of pathways (PP, FF and PK) from the three prototypes; (**c**) DXA LST *vs.* impedance index from all three prototype device pathways and (**d**) matrix plots of the impedance index between each pair of pathways (PP, FF and PK) from the three prototypes.

We observed strong correlations for impedance measurements between the prototype pathways and the corresponding reference IB electrical pathways (R = 0.93 [PP-01], 0.93 [PP-02], 0.93 [FF], 0.76 [PK], *p* < 0.0001) as shown in Figure 4a. Both PP and FF pathways supported reliable results with correlation coefficients >0.9 with respect to the IB device. Results from our pilot study encompassing repeated tests showed that the standard deviations of impedance values from the PP (Figure 3a left) and FF (Figure 3b) pathways were 1.43 Ω and 1.34 Ω, respectively. The FF (Figure 3b) pathway demonstrated an improved impedance distribution with higher linear correlation to reference devices relative to the PP pathway, owing to the smaller surface area of the electrodes (PP: 20 × 30 mm^2^, FF: 12.5 × 6 mm^2^) which minimized electrode flexing and provided more stable sensing pressure. We observed weaker correlations between the PK pathway and reference devices due to errors in measurement posture. In the PK pathway measurements, some subjects inadvertently positioned fingers from one hand on the ipsilateral leg which resulted in the creation of an unexpected current path adversely affecting total body resistance. We observed strong inter-pathway correlations among the three prototypes between PP and FF (R = 0.95), PP and PK (R = 0.85) and FF and PK (R = 0.85) as shown in Figure 4b. As stated previously, the PK pathway showed weaker correlations relative to the other pathways. The impedance index values, height^2^/Z, obtained from the prototypes were strongly correlated to the DXA LST measurements (R = 0.92 [PP], R = 0.93 [FF] and R = 0.82 [PK], *p* < 0.001). Such associations enabled more accurate assessments of body composition (Figure 4c,d).

To provide repeatability of measurement, we computed a saturation range and obtained the averaged data from the saturation window in data processing step. We measured impedance values three times for each pathway and evaluated repeatability of measurement by comparing coefficients of variation (CV) from multiple measurements as shown in Table 4. Each subject’s CV from three measurements for each pathway was averaged. CVs of 50 kHz impedance values at each pathway were less than 0.625% which indicated all values were controlled well with small variations (0.2% for PP, 0.3% for FF, 0.4% for PK pathway). The low frequency range, 5 kHz, showed higher CV and large standard variation in repeated measurement comparing to other frequencies because of high contact resistance. 

**Table 4:** Coefficients of variation (CV) of multiple frequency pathways (N = 560).

	PP			FF			PK		
	5 kHz	50 kHz	200 kHz	5 kHz	50 kHz	200 kHz	5 kHz	50 kHz	200 kHz
**CV (%)**	0.8 ± 1.1	0.2 ± 0.3	0.3 ± 0.7	1.1 ± 1.5	0.3 ± 0.4	0.3 ± 0.3	0.5 ± 0.7	0.4 ± 0.8	0.5 ± 0.5
**SD (Ohm)**	5.0	1.7	2.1	4.6	1.7	1.6	2.1	1.4	1.5

CV (%): Coefficient of Variation, SD (Ohm): Standard deviation of impedance measurements.

We derived a body composition equation with respect to the standard target data; DXA LST for skeletal muscle mass and FFM for body fat by feature selection followed by multivariate linear regression to estimate skeletal muscle mass and body fat. We tested assumptions before applying multiple regression method regarding lean mass. Normality of residuals is assumed (*p* = 0.482 > 0.05), and homoscedasticity of residuals is also assumed. The outcome of the model was S = 4.36 and R^2^ = 84.77%. The estimated body fat and skeletal muscle mass revealed no significant differences when compared to DXA (*p* > 0.728 (paired *t*-test), DXA fat = 29.45 ± 10.77 kg, estimated fat = 29.52 ± 12.53 kg; Table 5 and Table 6). The accuracy of body fat estimated from previous studies varied widely in terms of the standard error of estimate (SEE; 1.9%~5.0%) and the coefficient of determination, R^2^ (0.55~0.93). suggesting that our estimated results fit well compared to criterion standard data. In comparing these results to those from other commercial devices (HBF-214 (Omron) and S10 (InBody)), we observed a mean shift in the InBody S10 results (fat = 28.6 ± 9.2 kg, *p* < 0.0005). This was at odds with the manufacturer’s specifications, likely because DXA itself has some inherent errors ranging from 1.4 kg (2.7%) to 2.7 kg (4.5%) depending on the system type and measurement conditions [19,20]. 

**Table 5:** Correlation coefficients between the estimated and DXA results.

	PP-P1	PP-P2	FF	PK
Fat	0.92	0.91	0.92	0.90
SMM	0.96	0.95	0.95	0.94

DXA: Dual-energy X-ray absorptiometry, PP: Palm-to-Palm type prototype, FF: Finger-to-Finger type prototype, PK: Palm-to-Knee type prototype.

**Table 6:** Estimated values and differences relative to target values (paired *t*-tests).

Device	Body Fat (kg)			Skeletal Muscle Mass (kg)		
	Value	Difference	*p*-Value	Value	Difference	*p*-Value
DXA	29.5 ± 10.8	-	-	23.1 ± 6.7	-	-
PP	29.5 ± 12.5	−0.06 ± 4.4	0.728	23.0 ± 6.3	0.05 ± 2.4	0.602
FF	29.5 ± 9.8	−0.03 ± 4.4	0.863	23.1 ± 6.4	−0.01 ± 2.1	0.947
PK	29.7 ± 9.7	−0.26 ± 4.9	0.217	22.9 ± 5.8	0.12 ± 3.0	0.354

DXA: Dual-energy X-ray absorptiometry, PP: Palm-to-Palm type prototype, FF: Finger-to-Finger type prototype, PK: Palm-to-Knee type prototype.

**Figure 5:**
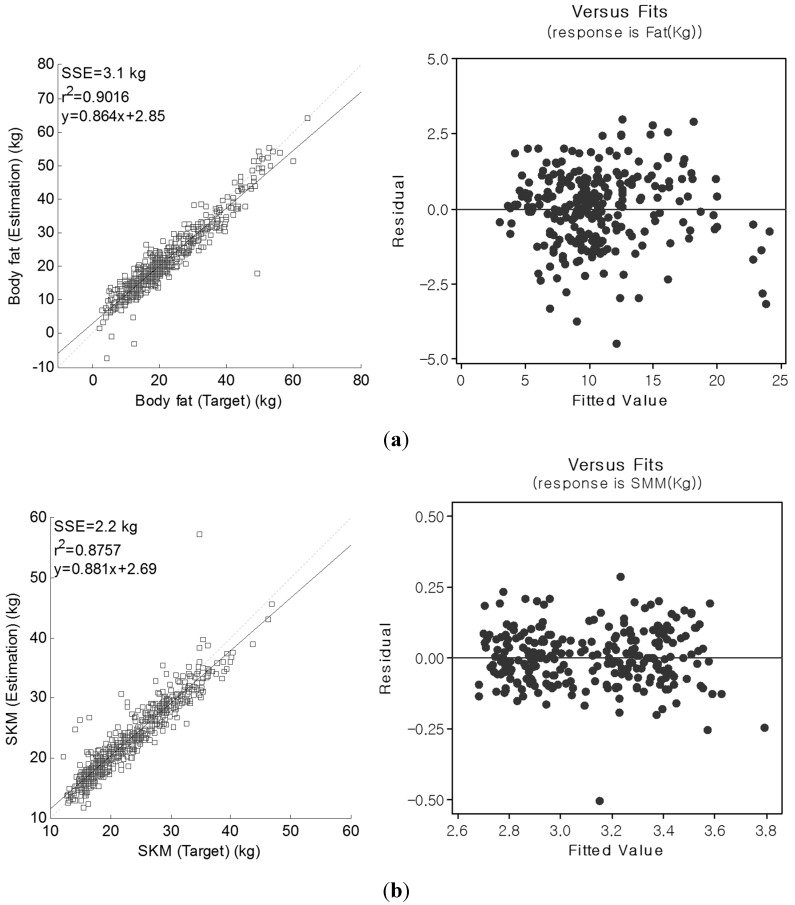
Regression and complementary residual *vs.* fitted plots of the PP pathway for (**a**) body fat (kg) and (**b**) skeletal muscle mass (kg).

We applied 2-fold cross validation to evaluate the regression result. Testing and validation subjects were randomly selected. Figure 5 shows regression and complementary residual *vs.* fitted plots of the PP pathway for body fat and skeletal muscle mass. The residual *vs.* fitted plots did not reveal any interactions for estimated body fat and skeletal muscle mass according to the range of body fat measures [7]. As seen in Figure 5, the differences between estimated and target values are equally distributed with respect to the mean values. This suggests that our estimated model showed reliable body fat and skeletal muscle mass, comparable with those from DXA whole-body composition. In some subjects, however, we observed large variations relative to DXA in the form of either under or overestimated body fat values. This was mainly observed in two subjects presenting with a low BMI value (19 Kg/cm^2^), and one subject demonstrating a high BMI value (35 kg/cm^2^), despite the impedance measurements from all three subjects being relatively small and resulting in large body fat and skeletal muscle mass estimation errors. In some subjects manifesting high BMI values (38.4 kg/cm^2^ and 40.1 kg/cm^2^), the skeletal muscle mass was overestimated, quite possibly owing to current propagation through the body fat as previously reported [8]. 

**Figure 6:**
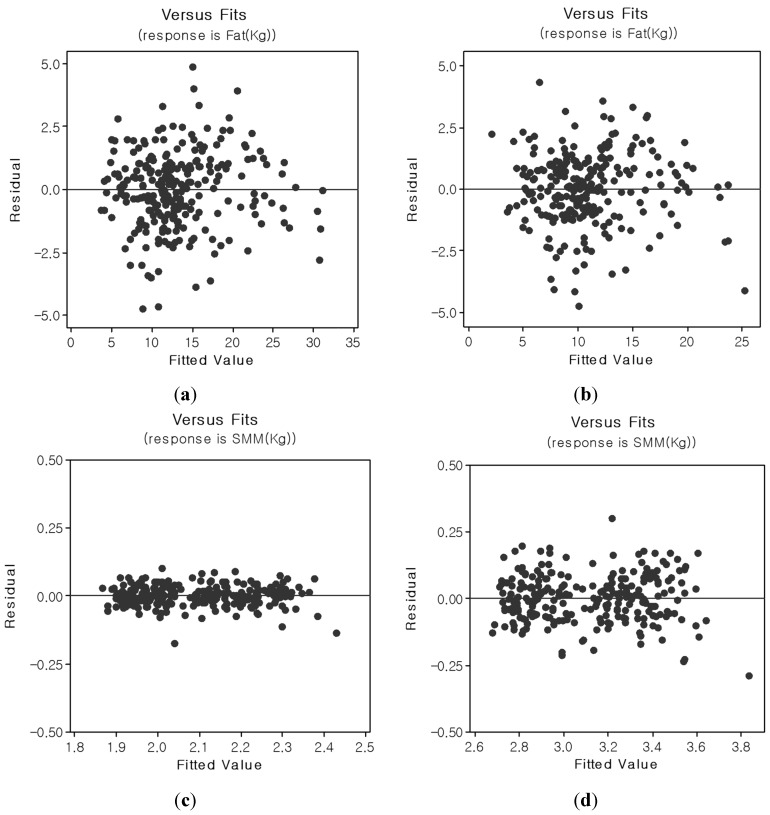
Residual *vs.* fitted plots for body fat and skeletal muscle mass (SKM) for the FF and PK pathways ((**a**) body fat [FF]; (**b**) body fat [PK]; (**c**) skeletal muscle mass [FF]; and (**d**) skeletal muscle mass [PK]).

Figure 6 shows residual *vs*. fitted plots between target and estimated body fat and skeletal muscle mass for the FF and PK pathways. The impedance distributions from the FF and PK devices paralleled those observed from the body composition analysis. A small estimation error of ±1.96 SD was noted for the FF pathway, and large estimation errors (>±1.96 SD) were observed for the PK pathway relative to PP. The PK pathway’s performance is strongly linked to the linearity of the current pathways of its device. Our future work will focus on modeling alternative current path problems for this device. 

To estimate body fat and skeletal muscle mass, subject data were analyzed on the basis of subgroups comprising three age groups, two sex groups, three BMI groups, and three race groups. The Bonferroni method is applied in their comparison. We tested one-way ANOVA for each subgroup analysis with one hypothesis. Our hypothesis was that there were no significant performance differences according to each subgroup. We observed significant variations in the sex and race sub-groups for body fat by computing the difference between target and estimated data as shown in Table 7 and Table 8. The sex sub-group analysis revealed that the male population tended to be underestimated compared to the female population, which was overestimated (error distribution mean ± SD, −1.22 ± 3.90 kg (males) and 1.03 ± 2.78 kg (females)). In the race sub-group, errors from the Asian group were negatively distributed, −0.95 ± 2.68 kg, relative to other races which were positively distributed (0.82 ± 3.87 kg for Caucasians and 1.43 ± 3.91 kg for African Americans). Skeletal muscle mass analysis based on sub-groups (Table 8) showed that there were tendencies to over/underestimate values according to sex, race, and BMI. The skeletal muscle mass in males was overestimated (0.70 ± 2.67 kg) compared to females (−0.45 ± 1.98 kg). The Asian population was slightly overestimated (0.41 ± 1.75 kg), but Caucasians were underestimated (−0.37 ± 2.64). Evaluating the high BMI group (obese population) alone demonstrated underestimated results (−0.74 ± 3.95 kg) compared to other BMI-based groups. These results parallel those reported by others in high BMI individuals. Our results showed that body fat was underestimated in the high BMI group which is a common finding in similar studies [8,21,22]. Owing to its high resistance, body fat may be inaccurately characterized as lean tissue especially in individuals with higher BMI values (>34) and thereby higher body fat levels. This can easily result in an underestimation of body fat in such individuals. 

**Table 7:** Statistical variables from subgroup analysis for estimated and target body fat.

Source	DF	Seq SS	Adj SS	Adj MS	F	P
Age group	2	15.93	16.62	8.31	0.81	0.445
Sex group	1	667.20	495.94	495.94	48.37	0.000
Race group	2	374.10	227.32	113.66	11.09	0.000
BMI group	2	55.41	55.41	27.71	2.7	0.068
Error	529	5423.77	5423.77	10.25		
Total	544	6536.42				

DF: Degrees of freedom, Seq SS: Sequential sum of squares, Adj SS: Adjusted sum of squares, Adj MS: Adjusted mean square, F: F statistic, P: Probability, BMI: Body Mass Index.

**Table 8:** Statistical variables from subgroup analysis for estimated and target skeletal muscle mass.

Source	DF	Seq SS	Adj SS	Adj MS	F	P
Age group	2	6.73	4.03	2.01	0.39	0.679
Sex group	1	171.68	138.84	138.84	26.76	0.000
Race group	2	52.39	45.97	22.99	4.43	0.012
BMI group	2	41.96	41.96	20.98	4.04	0.018
Error	529	2744.57	2744.57	5.188		
Total	544	3017.33				

DF: Degrees of freedom, Seq SS: Sequential sum of squares, Adj SS: Adjusted sum of squares, Adj MS: Adjusted mean square, F: F statistic, P: probability, BMI: Body Mass Index.

## 4. Conclusions/Outlook

In this work, we have proposed three different types of portable body composition analysis devices, where the impedance electrodes are located on the device cover, sides, and other configurations for integration into existing smartphone models. We evaluated the repeatability and reliability of these small electrodes by comparing their performance with other conventional commercial devices. In our evaluations, the portable BIA systems showed promise in estimating an individual’s body composition relative to measures generated by larger stationary BIA systems. Strong correlations were observed for impedance measured between the prototype pathways and corresponding InBody S10 electrical pathways (R = 0.93, *p* < 0.0001). Additionally, body fat was estimated without significant differences from criterion-standard DXA measurements (*p* > 0.728, DXA mean body fat, 29.45 ± 10.77 kg, and estimated body fat 29.52 ± 12.53 kg). The results suggest that overall estimation was comparable with the DXA system. However, subgroup analyses showed small over- or underestimations of body fat in certain discrete groups. The estimated results varied according to user profile. A limitation of the proposed devices is that because they are designed to be integrated and used with existing smartphone platforms, this makes the devices available to a select and affluent population base that can afford smartphones. However, to our knowledge, the number of individuals who can afford such devices is increasing yearly as referenced by the annual sale of smartphones worldwide [23]. 

Future developments entail extending this work to younger and elderly populations. Many reports emphasize that children and elderly people have different body composition compared to healthy adults [24,25,26]. The importance of body composition analysis is younger individuals is vital given that childhood obesity is a severe problem in the United States. We aim to address this by developing BIA algorithms for children (ages 10–17 years). Additionally, other portable devices such as wearables and wrist- and patch-type BIA devices will be researched. Our current study for verifying the feasibility of portable body composition systems in an adult population and validating the results against criterion-standard BIA devices has also provided insights into scaling such devices in other portable form factors. 

## References

[1] InBody Body Composition Analyzer 720. http://www.inbody.com/global/product/InBody720.aspx.

[2] Omron Weight Management Device BF306. http://www.omron-healthcare.com/eu/en/our-products/weight-management/bf306.

[3] Withings Smart Body Analyzer. http://www.withings.com/eu/smart-body-analyzer.html.

[4] Deurenberg P., Tagliabue A., Schouten F.J. (1995). Multi-frequency impedance for the prediction of extracellular water and total body water. Br. J. Nutr..

[5] Kyle U.G., Bosaeus I., de Lorenzo A.D., Deurenberg P., Elia M., Gómez J.M., Heitmann B.L., Kent-Smith L., Melchior J.-C., Pirlich M. (2004). Bioelectrical impedance analysis—part I: Review of principles and methods. Clin. Nutr..

[6] Kyle U.G., Bosaeus I., de Lorenzo A.D., Deurenberg P., Elia M., Gómez J.M., Heitmann B.L., Kent-Smith L., Melchior J.-C., Pirlich M. (2004). Bioelectrical impedance analysis—part II: Utilization in clinical practice. Clin. Nutr..

[7] O’Connor D.P., Mahar M.T., Laughlin M.S., Jackson A.S. (2011). The Bland-Altman method should not be used in regression cross-validation studies. Res. Q. Exerc. Sport.

[8] Baumgartner R.N., Ross R., Heymsfield S.B. (1998). Does adipose tissue influence bioelectric impedance in obese men and women?. J. Appl. Physiol..

[9] Kotler D.P., Burastero S., Wang J., Pierson R.N. (1996). Prediction of body cell mass, fat-free mass and total body water with bioelectrical impedance analysis: Effects of race, sex, and disease. Am. J. Clin. Nutr..

[10] Kyle U.C., Genton L., Karsegard L., Slosman D.O., Pichard C. (2001). Single prediction equation for bioelectrical impedance analysis in adults aged 20–94 years. Nutrition.

[11] Deurenberg P., van der Kooy K., Leenen R., Westrate J.A., Seidell J.C. (1991). Sex and age specific prediction formulas for estimating body composition from bioelectrical impedance: A cross-validation study. Int. J. Obes..

[12] Heitmann B.L. (1990). Prediction of body water and fat in adult danes from measurement of electrical impedance. A validation study. Int. J. Obes..

[13] Xu L., Cheng X., Wang J., Cao Q., Sato T., Wang M., Zhao X., Liang W. (2011). Comparisons of body-composition prediction accuracy: A study of 2 bioelectric impedance consumer devices in healthy Chinese persons using DXA and MRI as criteria methods. J. Clin. Densitom..

[14] Ramel A., Geirsdottir O.G., Arnarson A., Thorsdottir I. (2011). Regional and total body bioelectrical impedance analysis compared with DXA in Icelandic elderly. J. Clin. Nutr..

[15] Piccolia A., Pastoria G., Codognottoa M., Paolia A. (2007). Equivalence of information from single frequency *v.* bioimpedance spectroscopy in bodybuilders. Br. J. Nutr..

[16] Wouter D., Lichtenbelt V.M., Hartgens F., Vollaard N., Ebbing S., Kuipers H. (2004). Body composition changes in bodybuilders: A method comparison. Med. Sci. Sports Exerc..

[17] Seo S. A Review and Comparison of Methods for Detecting Outliers in Univariate Data Sets. Master’s Thesis.

[18] World Health Organization Global Recommendations on Physical Activity for Health. http://apps.who.int/iris/bitstream/10665/44399/1/9789241599979_eng.pdf.

[19] Haarbo J., Gotfredsen A., Hassager C., Christiansen C. (1991). Validation of body composition by dual energy X-ray absorptiometry (DEXA). Clin. Physiol..

[20] Svendsen O.L., Haarbo J., Hassager C., Christiansen C. (1993). Accuracy of measurements of body composition by dual-energy X-ray absorptiometry *in vivo*. Am. J. Clin. Nutr..

[21] Pateyjohns I.R., Brinkworth G.D., Buckley J.D., Noakes M., Clifton P.M. (2006). Comparison of three bioelectrical impedance methods with DXA in overweight and obese Men. Obesity.

[22] Coppini L.Z., Waitzberg D.L., Campos A.C. (2005). Limitations and validation of bioelectrical impedance analysis in morbidly obese patients. Curr. Opin. Clin. Nutr. Metab. Care.

[23] Gartner Report: Gartner Says Smartphone Sales Surpassed One Billion Units in 2014. http://www.gartner.com/newsroom/id/2996817.

[24] Kim J., Shen W., Gallagher D., Jones A., Wang Z., Wang J., Heshka S., Heymsfield S.B. (2006). Total-body skeletal muscle mass: Estimation by dual-energy X-ray absorptiometry in children and adolescents. Am. J. Clin. Nutr..

[25] Baumgartner R.B., Heymsfield S.B., Lichtman S., Wang J., Pierson R.N. (1991). Body composition in elderly people: Effect of criterion estimates on predictive equations. Am. J. Clin. Nutr..

[26] Roubenoff R., Baumgartner R.N., Harris T.B., Dallal G.E., Hannan M.T., Economos C.D., Stauber P.M., Wilson P.W.F., Kiel D.P. (1997). Application of bioelectrical impedance analysis to elderly populations. J. Gerontol..

